# Temporal changes in the Swiss flora: implications for flower-visiting insects

**DOI:** 10.1186/s12862-022-02061-2

**Published:** 2022-09-15

**Authors:** Stefan Abrahamczyk, Michael Kessler, Tobias Roth, Nico Heer

**Affiliations:** 1 Nees-Institute for Biodiversity of Plants, University of Bonn, Meckenheimer Allee 170, 53115 Bonn, Germany; 2 Systematic and Evolutionary Botany, University of Zurich, Zollikerstr. 107, 8008 Zurich, Switzerland; 3 Department of Environmental Sciences, Zoology, University of Basel, Basel, Switzerland; 4 Ecological Consultancy, Planning and Research, Hintermann and Weber AG, Reinach, Switzerland; 5 Stuttgart State Museum of Natural History, Rosenstein 1, 70191 Stuttgart, Germany

**Keywords:** Evenness, Floristic change, Flower colour, Flower type, Functional diversity, Pollination, Reproductive ecology, Self-incompatibility

## Abstract

**Background:**

Local floristic diversity has massively decreased during the twentieth century in Central Europe even though in the 1990s diversity began increasing again in several regions. However, little is known whether this increase is equally distributed among plant groups with different reproductive traits.

**Methods:**

Our study is based on data of the Swiss Biodiversity Monitoring Program. In this program, plant species occurrence is recorded since 2001 in 450 regularly distributed 1 km^2^ study sites. For all 1774 plant species registered in the study, we researched data on flower/pseudanthium type and colour, reproductive system, and groups of flower visitors. We then tested whether temporal changes in species frequency were equally distributed among species with different trait states.

**Results:**

Species richness and functional richness significantly increased in the study sites while functional evenness decreased. The frequency of wind-pollinated species increased more strongly than that of insect-pollinated species. Further, the frequency of species with simple, open insect-pollinated flowers and pseudanthia visited by generalist groups of insects increased slightly more strongly than the frequency of species with complex flowers visited by more specialized groups of flower visitors. Additionally, the frequency of self-compatible species increased significantly more than that of self-incompatible species. Thus, the overall increase in local plant species richness in Switzerland is mostly driven by wind- and generalist insect-pollinated, self-compatible species. In contrast, species with complex flowers, which are essential for specialized groups of flower visitors and species with self-incompatible reproductive systems profited less.

**Conclusions:**

Our study thus emphasizes the need to consider functional traits in the planning and monitoring of conservation activities, and calls for a special focus on plant species with specialized reproductive traits.

**Supplementary Information:**

The online version contains supplementary material available at 10.1186/s12862-022-02061-2.

## Background

Habitat loss and fragmentation due to agricultural land use change and urbanization as well as climate change have led to a drastic decrease in plant and pollinator species richness and massive changes in assemblage composition over large parts of Europe since the beginning of the twentieth century [1, 12, 19, 21, 44]. These declines were most pronounced before the 1990ies [12]. Since then, changes in conservation policies (e.g., [6, 9]) have slowed down the decline in species richness and even reversed them for some groups on smaller spatial scales, while homogenization of community often continued [8, 12]. Especially plant species from nutrient-poor, open habitats as well as cold-adapted species continue to decline whereas competitive, tall-growing, thermophilous species from nutrient-rich habitats have increased [38, 42–44]. On the pollinator side, Europe has experienced much publicized collapses of insect populations over the last few decades [45, 46]. Especially insect species with a specialised breeding or feeding behaviour, including many long-tongued species have declined drastically (e.g., [5, 18, 33]). Often, naturally rare plant and pollinator species have become even rarer whereas naturally abundant species and thermophilous species have increased in abundance [5, 18, 19, 35]. This decrease of the majority of species and increase of a small proportion of species has led to a biological homogenization of many habitat types [12, 35]. However, also regions with increasing floristic diversity have shown constant or even decreasing functional increasing and increasing evenness [8, 19, 39].

In most studies cited above, changes of plant functional richness and evenness were analysed based on changes of mean ecological Ellenberg indicator values [19, 42–44] in the community and/or based on proportional changes of abundance or cover of species with different vegetative traits such as life form or growth height. In contrast, few studies have included reproductive traits into analyses on biological homogenization [1, 12]. These studies mostly applied trait data such as flower dependence on insect pollination vs. wind pollination, or data on groups of flower visitors, and have shown that specialized plant-pollinator interactions have declined most strongly. However, these studies neither used information on flower morphology and coloration, nor data on self-compatibility vs. self-incompatibility to study homogenization of functional traits. Functional plant traits, however, are much more useful to understand whether plant species are more or less important for the ecology of different groups of flower visitors than just presence-absence data on flower visitor groups. For instance, specialized pollinators such as bumblebees and other long-tongued bees may occasionally use open flowers for collecting nectar but are dependent on the availability of high-rewarding lip and flag flowers [49]. On the other hand, complex flowers may be occasionally visited and pollinated by less specialized groups of flower visitors but are dependent on pollination by specialized pollinators such as long-tongued bees for successful seed production [3, 11]. Thus, these plant species suffer more from a decrease in species richness and abundance of their pollinators, as has happened in large parts of Europe [4, 7, 14].

Additionally, it has been hypothesized that self-incompatible species suffer more from habitat fragmentation and pollinator declines than self-compatible species [2, 41]. On the other hand, many self-incompatible plant species are highly important for flower-visiting insects (e.g., [31, 37, 49]). Sparse data exist already on population changes of individual self-incompatible species due to pollinator limitation and fragmentation (e.g. [2, 10, 13, 24]). These data largely support that self-incompatible species are under severe pressure but analyses on community level are still missing.

To investigate the floristic development in relation to different reproductive traits during the last two decades, we used data on changes in floristic composition of 450 sites of the Biodiversity Monitoring Program of Switzerland and added information on three reproductive traits (flower/pseudanthium type and colour, and reproductive system) as well as information on flower visitor groups. Bühler and Roth [8] showed that floristic species richness has increased whereas beta diversity has decreased in Swiss grasslands since 2001. Additionally, specialized insect species have continued to decline [48, 50]. Based on the increases of local plant species richness in combination with decreasing diversity of specialized insects, we formulated two hypotheses:

1. The frequency of wind-pollinated and open-flowered plant species—typically visited by unspecialized groups of flower visitors—has increased more strongly than the frequency of species with complex flowers visited by more specialized groups of flower visitors.
2. The frequency of self-compatible plant species have increased more strongly than the frequency of self-incompatible species.

## Results

**Fig. 1:**
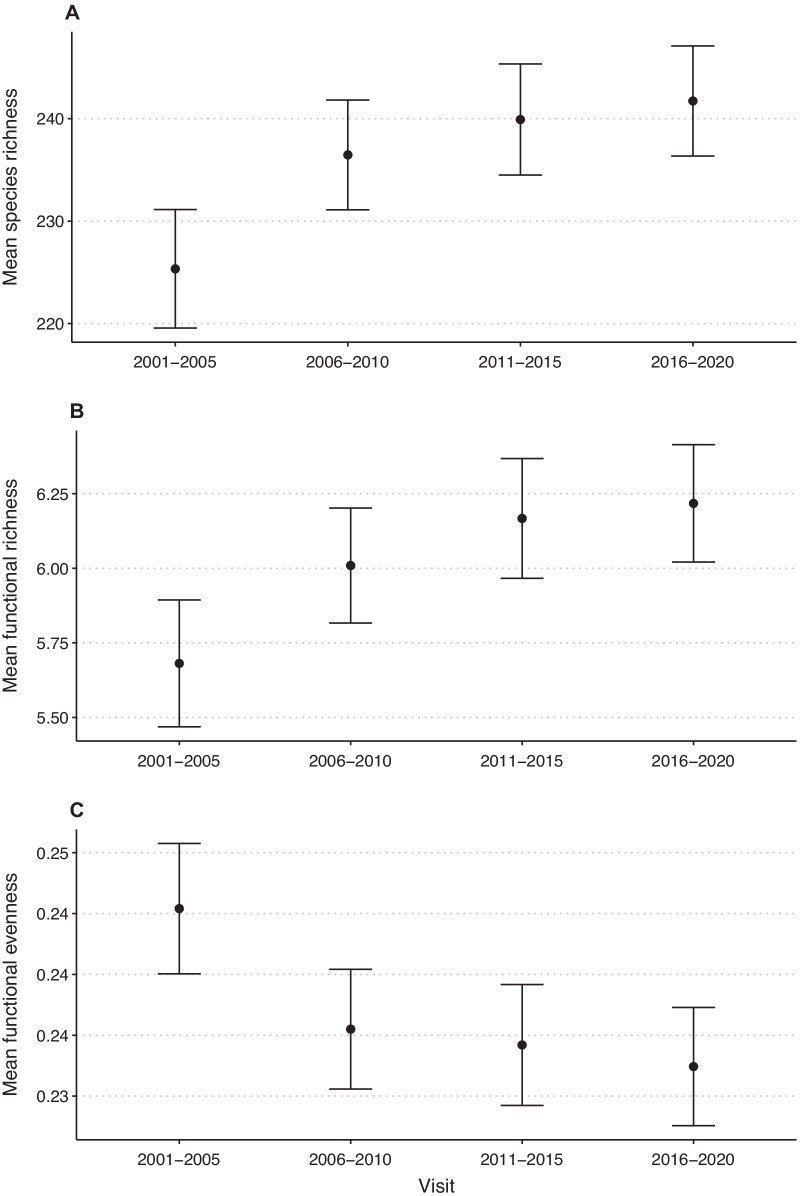
Changes in mean (**A**) plant species richness, (**B**) functional richness of reproductive traits, and (**C**) functional evenness of reproductive traits at the study sites of the Swiss Biodiversity Monitoring Program in four time periods between 2001 and 2020. Centroids are means of the 448 study sites, and brackets the 95% confidence intervals

Since the year 2001, 1774 species of seed plants (Additional file 1: Table S2) have been recorded in the 448 investigated study sites of the Biodiversity Monitoring Program of Switzerland. For 428 (24.1%) species we found a negative frequency trend, 86 (4.8%) species did not show a significant change in frequency, and 1260 (71.0%) species increased in frequency. Mean positive frequency trends (+ 19% per 5 years) were larger than mean negative trends (− 11% per 5 years). At the study sites, mean species richness increased from 225 at the first visit to 242 at the fourth visit (Fig. 1a). Mean functional richness of reproductive traits increased in parallel (from 5.68 at the first visit to 6.22 at the fourth visit; Fig. 1b). Functional evenness of reproductive traits, however, decreased from 0.245 at the first visit to 0.232 at the fourth visit (Fig. 1c).

**Fig. 2:**
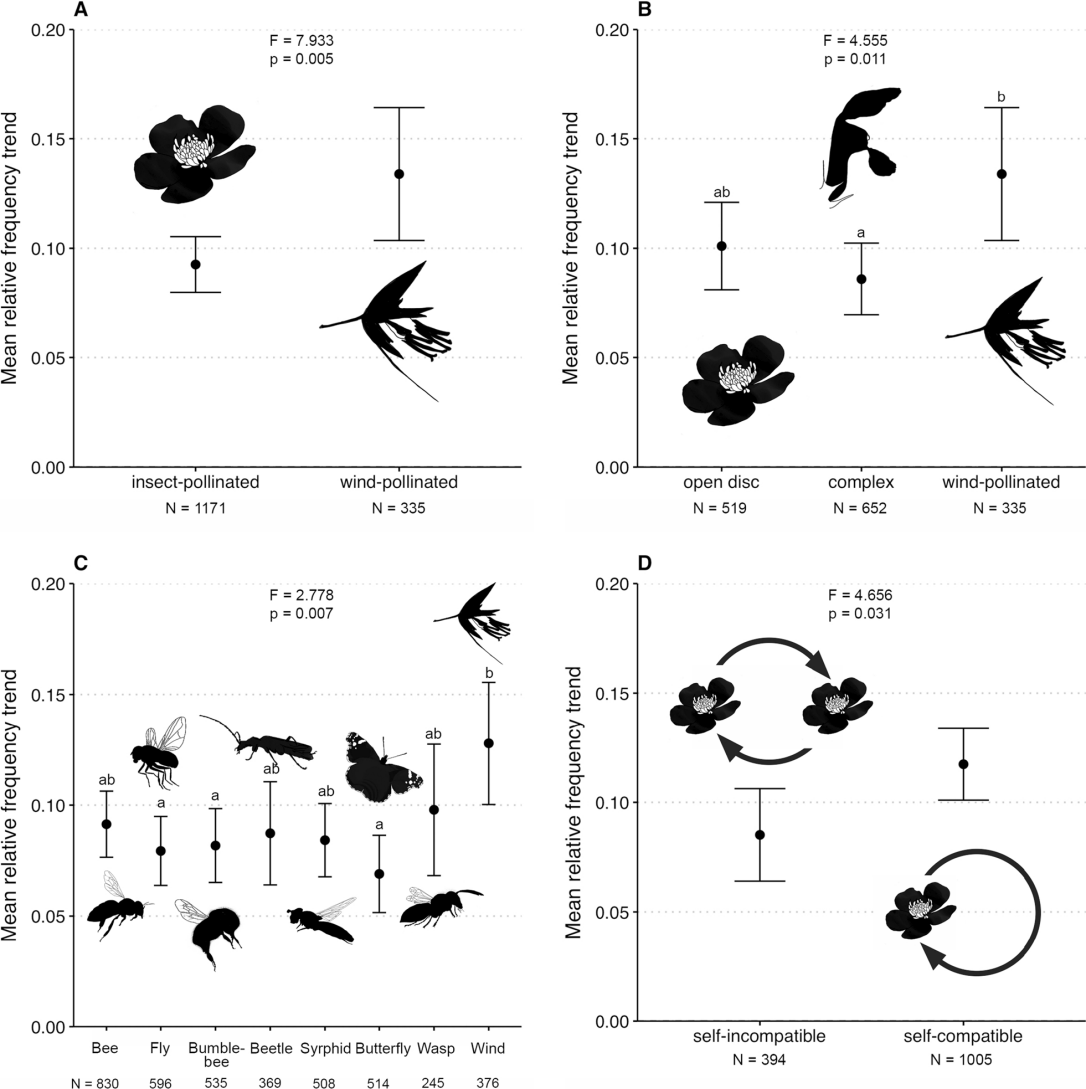
Changes in mean frequencies over the 20-year study period of (**A**) insect vs. wind-pollinated species, (**B**) open saucer-shaped vs. complex and wind-pollinated flower/pseudanthium types, (**C**) plant species utilized by different groups of flower visitors, and (**D**) self-compatible vs. self-incompatible plant species. Centroids are means of the 448 study sites, and brackets the 95% confidence intervals. Drawings by S. Kessler

The relative frequency of species visited by insects increased significantly less than species pollinated only by wind (Fig. 2a). Separating between simple insect-pollinated flowers (saucer-shaped flowers), complex flowers, and wind-pollinated flowers revealed only significant difference in relative frequency increase between complex and wind-pollinated flowers (Fig. 2b). Divided onto flower/pseudanthium types, we did not find significant differences in the increases of mean species relative frequency (Additional file 1: Fig. S2a). However, species with green and brown flowers/pseudanthia increased significantly more strongly than species with violet flowers/pseudanthia (Additional file 1: Fig. S2b). Additionally, plant species visited by bumblebees, butterflies, or flies increased significantly less strongly than plant species pollinated by wind (Fig. 2c). Finally, the mean relative trends of self-compatible plant species increased more strongly than the mean relative frequency trends of self-incompatible species (Fig. 2d).

The AIC showed that a linear model without interactions among variables was preferable. In this model, only self-(in)compatibility (p = 0.019) significantly explained variation in species frequencies while pollination type (insect vs. wind, p = 0.192) was not significant.

## Discussion

In this study we analysed changes of local species richness and reproductive trait diversity as well as functional richness and evenness in Swiss plant assemblages since 2001 and their implications for different groups of flower visitors. We found that plant species richness increased on average by 7.5% (from 225 to 242 species per site), possibly due to a combination of environmental and nature protection activities leading to a reduction in the intensity of agricultural practice [6, 9], increasing abundance of neophytic species [28], and climate change effects, especially in mountains where they allow the upward shift of species [36, 40]. Additionally, a slight increase in floristic knowledge of the field botanists cannot be excluded. However, it should be borne in mind that the starting point of this positive trend probably corresponds to the historical low in plant and flower visitor species richness.

Increases of local plant as well as flower visitor species richness over the last few decades have also been observed in other regions of Central Europe [19, 39]. On the other hand, the parallel decrease of functional evenness of reproductive traits indicates that the increase in species richness is not equally distributed among functional groups in the Swiss flora. Wind-pollinated species (mostly grasses) with green and brown flowers/pseudanthia increased much more strongly than insect-pollinated species. Among insect-pollinated species, plant species with simple saucer-shaped, mostly white or yellow flowers/pseudanthia, visited by a large number of different, less specialized groups of flower visitors increased slightly more than plant species with more complex flowers, such as mostly violet lip or flag flowers. As a consequence, functional evenness of reproductive plant traits has decreased strongly since 2001. The invasion of wind-pollinated species, mostly grasses, bears negative consequences for the entire assemblage of flower visitors. However, polylectic, short-tongued groups of flower visitors may benefit more from the increase of open flowers/pseudanthia whereas long-tongued species and specialists of complex flowers may less be able to increase their populations (e.g., [5, 15, 33]). This pattern is of course group- and region-specific: for example, bee diversity has increased in Great Britain and the Netherlands, and hoverfly diversity has risen in Belgium since 1990 [12]. However, these increases are partly driven by the rise of thermophilous species and the assemblages are still depauperate and functionally homogeneous. For Switzerland these tendencies are reflected in the butterfly fauna [48, 50].

Even more pronounced than the increase of plant species adapted to pollination by wind or generalist insect groups is the strong increase of self-compatible plant species in contrast to self-incompatible species. This tendency may be problematic because the vast majority of self-incompatible species (80.1%) is visited by insects and dependent on their pollination activity. If this group of plants recovers less well than self-compatible species, this has severe consequences for their often highly adapted flower visitors and vice versa: To attract sufficient pollinators, many of the insect-pollinated, self-incompatible species provide conspicuous, mostly complex (55.9%) flowers, producing large amounts of pollinator reward (mostly nectar and pollen). These complex flowers of, for example *Linaria* or *Phyteuma* provide essential food resources for long-tongued bees and bumblebees and cannot be utilized by non-adapted groups of flower visitors. Additionally, several self-incompatible species/genera are essential for the resource availability for entire pollinator assemblages during parts of the year. For example, the genus *Salix* represents a keystone resource for flower visitors in early spring when few other species are flowering [31]. Further, several self-incompatible genera/species, such as *Campanula* or *Lysimachia* are the only source of pollen and in case of *Lysimachia* also floral oil for many oligolectic bee species [49]. These oligolectic bee species again are highly efficient pollinators for their food plant species since they specifically search for their plants and deposit mostly intra-specific pollen [49]. Unfortunately, this narrow dependency limits the potential for a recovery of both groups of species and may give a hint why self-compatible species have increased more strongly. Ultimately, we are faced with a situation where specialized interdependent pollination systems are decreasing, probably disrupting long-evolved functional relationships. Once these are broken up, the recovery of both the plant species and their specialized flower visitors may be greatly inhibited.

## Conclusions

Our study underlines that the simple increase of species richness should not be interpreted as a pure positive development [22]. In the Swiss flora, the increase of diversity is mostly driven by an increase of self-compatible, wind-pollinated, and open-flowered species. Future studies should investigate this development based on species abundance instead of presence-absence data, such as in our study. For pollinators dependent on the availability of complex flowers the increase of wind-pollinated and generalist insect-pollinated species neither improves the availability of food nor does it improve the situation for self-incompatible plant species with complex flowers dependent on specialized pollinators. Thus, our study stresses the importance of supporting activities for populations of plant species with complex flower types and a self-incompatible reproductive system. Further, functional reproductive traits should be incorporated in the planning and monitoring of conservation activities. As a positive effect, these conservation activities will also support many highly endangered species of flower visitors.

## Material and methods

### Study design and sampling

We analyzed presence-absence data of seed plant species from the Biodiversity Monitoring Program of Switzerland (BDM, www.biodiversitymonitoring.ch; [47]. In this monitoring program, plant surveys are conducted in 450 1 km^2^ study sites which are equally spaced in a grid over Switzerland, representing nearly all elevation zones and habitat types. At each study site, a transect of 2.5 km length and 5 m width (a total of 12,500 m^2^) is surveyed within the 1 km^2^ grid cell. The transects follow existing trails, preferably near the diagonal of the grid cell [32]. Their course was defined in a way that they lie completely within the grid cell. The use of the existing trail network had practical reasons. Consequently, typical plant species of standing waters, marshes, and swamps are less fully represented (unpublished data). Vegetation surveys are carried out by qualified botanists following standardized field-protocols (www.biodiversitymonitoring.ch/index.php/en/methology/diversity-in-landscapes). To encompass a large variation in flowering phenologies, sampling took place in two defined time periods, once in spring and once in summer. Only transects (or segments thereof) in the alpine zone, having a short vegetation period, were visited once. We analyzed data of four study periods from 2001 to 2020 (2001–2005, 2006–2010, 2011–2015, 2016–2020). Each site was visited once every 5 years because in each year approximately a fifth of the sites were surveyed. In some years individual sites could not be visited, e.g. due to problems of accessibility. Thus, of the 450 study sites, 396 were visited four times and 52 three times. We excluded two study sites with only two visits.

### Reproductive traits

We extracted data on flower/pseudanthium type and color from www.biolflor.de [16] for all species. In this data base, ten flower/pseudanthium types are listed: bell, trap, lip, tube (including funnel-shaped), saucer-shaped (= disc), flag, Stielteller, ray, water, and wind. We categorized Asteraceae with both tube and ray flowers, e.g. *Leucanthemum vulgare* agg. as tube flowers because their ray flowers are sterile in many species [23] and thus function only to attract flower visitors. Eight main flower/pseudanthium colors are listed in www.biolflor.de: blue, brown, yellow, green, red (including rose), violet, purple and white. We used all of these categories but fused violet and purple due to large overlap of the categorization in the database. We categorized Asteraceae with both tube and ray flowers with different colors based on the color of the tube flowers. For species not listed in www.biolflor.de, we categorized flower/pseudanthium type and color based on photos and descriptions in Lauber and Wagner [26]. Data of flower/pseudanthium type and color exist for 100% of the species.

We took data on self-(in)compatibility mostly from www.biolflor.de [16]. For this trait, Durka [16] collected data mostly from historic publications (e.g., [17, 20]). He divided the species into four categories (self-compatible, mostly self-compatible, mostly self-incompatible, self-incompatible) based on the historic descriptions (W. Durka, pers. comm.). Due to the subjectivity when categorising descriptions and to include the natural variability in the degree of self-(in)compatibility between plant individuals and populations of the same species, we decided to merge self-compatible and mostly self-compatible as well as mostly self-incompatible and self-incompatible species to one category each (largely self-compatible and largely self-incompatible species). Species categorized in www.biolflor.de as obligately apomictic as well as species categorized as facultatively or obligately autogamous were included under self-compatible species. Dioecious species were categorized as self-incompatible. We further searched www.scholargoogle.de for data on reproductive systems of species not included into www.biolflor.de or with missing data using the search terms “compatible”, “selfing” or “apomixis” (only in the genera *Hieracium, Potentilla, Ranunculus*, and *Sorbus*) and the Latin species name. We only included information if self-(in)compatibility or obligate apomixis was shown experimentally. If seed set was reduced by > 50% in geitonogamy treatments in comparison to xenogamy treatments, we categorized a species as self-incompatible. In total we found data on self-(in)compatibility for 79.2% of the species (Additional file 1: Table S1).

### Flower visitors

Building on the data set of Abrahamczyk et al. [1] we collected data on the groups of flower visitors for all plant species from the literature. We differentiated between seven diurnal groups of flower visitors: bees, bumblebees, wasps, butterflies, syrphid flies, other flies and beetles (Additional file 1: Table S2). Additionally, we registered whether a species is known to be wind pollinated. Since only a single species in our data set is water-pollinated (*Lemna minor*) we excluded this species from the analyses. Additional data was mostly collected from [23, 25]. If no information was available in these books, we conducted a literature search in www.scholargoogle.de using the search terms “pollinator” in combination with the Latin name of the species. In total we found data on flower visitors/wind pollination for 85.2% of the species.

### Data analyses

First, we calculated temporal trends in the number of occupied sites (i.e. sites with species occurrence) for each of the 1774 species recorded in the dataset. As every study site is surveyed once every 5 years, we calculated the number of sites a species was present in each of the four 5-year time periods (2001–2005, 2006–2010, 2011–2015, 2016–2020). To obtain relative frequencies for each species we divided the number of occupied sites per period by the number of occupied sites in the period where the species first appeared. For each species, we calculated the slope of a linear regression with the relative frequency as dependent variable and period as predictor variable. The estimated slope was our measure of the species’ frequency trend given as the change in % per 5 years. These species’ trends were then linked with corresponding reproductive traits as described above.

For every single trait state we calculated the mean and confidence intervals of all species’ trends. Pairwise differences in means were investigated using one-way analysis of variance (ANOVA) and the post-hoc Tukey’s HSD (honestly significant difference) test. The three neophytes *Eragrostis pilosa* agg., *Senecio inaequidens* and *Cotoneaster divaricatus* agg. were excluded from the analyses, because they stood out as extreme outliers since they represent ongoing invasion and thus showed the highest relative temporal trends exceeding the next following species by two- to fourfold. The number of neophytes in our data set contains only 3% and 0.7% of the species are listed as invasive.

Before forming the final model with multiple reproductive traits as explanatory variables to explain variation in relative species frequencies, we checked correlations between traits using non-metric multidimensional scaling [30]. The results of the NMDS (Additional file 1: Fig. S1) document that the floral traits flower/pseudanthium type and color, and the different groups of flower visitors are highly correlated. Further, most insect-pollinated plant species are visited by a large variety of flower visitor groups. Only the traits wind-pollination and self-(in)compatibility are clearly separated from the insect-pollinated flower/pseudanthium types and colors. To reduce collinearity and limit the number of variables, we decided to conduct linear models with independent variables to explain variation in relative species frequencies, either pollination type (insect vs. wind) or self-(in)compatibility. All models were once calculated with interactions among variables and once without. The best fitting models were chosen based on AIC (Akaike Information Criterion).

Lastly, we estimated functional richness and evenness using the R package “ecospace” version 1.4.2 [29]. The package provides an easy to use wrapper function using principal coordinates analysis (PCoA) to return PCoA axes, which are then used to compute multidimensional functional diversity measures. We incorporated the traits flower/pseudanthium type and colour, and self-(in)compatibility. Since an ordination method (PCoA) is applied to calculate distances between species, we were able to incorporate categorical variables for the calculation of functional richness and evenness. When the species-by-species distance matrix could not be represented in a Euclidean space, we chose the lingoes-correction method, as recommended by Legendre and Anderson [27]. We conducted all analyses in R version 4.0.3 [34].

## Supplementary Information

**Additional file 1.** **Table S1:** Flower visitor groups and self-compatibility for the species found in the Biodiversity Monitoring Program of Switzerland. **Table S2:** Species observations on the 448 transects of the Biodiversity Monitoring Program of Switzerland and their relative frequencies. **Figure S1:** NMDS graphic showing relations between different reproductive trait states. **Figure S2:** Mean relative changes of different states of (A) blossom type and (B) blossom colour including the results of the ANOVAs and the Tukey tests. Note that in B the Tukey tests did not find significant differences.

## Data Availability

Data is available at www.zenodo.org. For requesting the original data contact TR (roth@hintermannweber.ch).

## Abbreviations

AIC: Akaike information criterion
ANOVA: Analysis of variance
BDM: Biodiversity monitoring program of Switzerland
NMDS: Non-metric multidimensional scaling
PCoA: Principal coordinates analysis
Tukey’s HSD: Honestly significant difference
