# Supplementary material for: Temporal changes in the Swiss flora: implications for flower-visiting insects
Source: BMC Ecol Evol. 2022 Sep 15;22:109. doi: 10.1186/s12862-022-02061-2 (PMC9479241; doi:10.1186/s12862-022-02061-2)
Supplement: Supplementary file 1 — Additional file 1. Table S1: Flower visitor groups and self-compatibility for the species found in the Biodiversity Monitoring Program of Switzerland. Table S2: Species observations on the 448 transects of the Biodiversity Monitoring Program of Switzerland and their relative frequencies. Figure S1: NMDS graphic showing relations between different reproductive trait states. Figure S2: Mean relative changes of different states of (A) blossom type and (B) blossom colour including the results of the ANOVAs and the Tukey tests. Note that in B the Tukey tests did not find significant differences. [file 12862_2022_2061_MOESM1_ESM.docx]

**Supplement**

**Temporal changes in the Swiss flora – implications for flower-visiting insects**

*Stefan Abrahamczyk, Michael Kessler, Tobias Roth, Nico Heer*

**Table S1:** Flower visitor groups and self-compatibility for the species found in the Biodiversity Monitoring Program of Switzerland.

Deposited at [www.zenodo.org](http://www.zenodo.org): doi:10.5281/zenodo.6566791.

**Table S2:** Species observations on the 448 transects of the Biodiversity Monitoring Program of Switzerland and their relative frequencies.

Deposited at [www.zenodo.org](http://www.zenodo.org): doi:10.5281/zenodo.6566835.

**Figure S1:** NMDS graphic showing relations between different reproductive trait states.

**Figure S2:** Mean relative changes of different states of (A) blossom type and (B) blossom colour including the results of the ANOVAs and the Tukey tests. Note that in B the Tukey tests did not find significant differences.
